# Electromechanical Behavior of Chemically Reduced Graphene Oxide and Multi-walled Carbon Nanotube Hybrid Material

**DOI:** 10.1186/s11671-015-1216-5

**Published:** 2016-01-05

**Authors:** Abderrahmane Benchirouf, Christian Müller, Olfa Kanoun

**Affiliations:** Measurement and Sensor Technology, Technische Universität Chemnitz, 09126 Chemnitz, Germany

**Keywords:** Multi-walled carbon nanotubes, Graphene oxide, Chemical reduction, Piezoresistivity, Strain sensor

## Abstract

In this paper, we propose strain-sensitive thin films based on chemically reduced graphene oxide (GO) and multi-walled carbon nanotubes (MWCNTs) without adding any further surfactants. In spite of the insulating properties of the thin-film-based GO due to the presence functional groups such as hydroxyl, epoxy, and carbonyl groups in its atomic structure, a significant enhancement of the film conductivity was reached by chemical reduction with hydro-iodic acid. By optimizing the MWCNT content, a significant improvement of electrical and mechanical thin film sensitivity is realized. The optical properties and the morphology of the prepared thin films were studied using ultraviolet-visible spectroscopy (UV-Vis) and scanning electron microscope (SEM). The UV-Vis spectra showed the ability to tune the band gap of the GO by changing the MWCNT content, whereas the SEM indicated that the MWCNTs were well dissolved and coated by the GO. Investigations of the piezoresistive properties of the hybrid nanocomposite material under mechanical load show a linear trend between the electrical resistance and the applied strain. A relatively high gauge factor of 8.5 is reached compared to the commercial metallic strain gauges. The self-assembled hybrid films exhibit outstanding properties in electric conductivity, mechanical strength, and strain sensitivity, which provide a high potential for use in strain-sensing applications.

**Electronic supplementary material:**

The online version of this article (doi:10.1186/s11671-015-1216-5) contains supplementary material, which is available to authorized users.

## Background

In the last decade, the increasing trend towards flexible electronic devices emphasizes their attractive perspective in numerous applications. Essential components in such multifunctional devices are sensors, which should be flexible, scalable, sensitive, and robust for integration. Due to their promising electrical and mechanical properties, carbon nanotubes (CNTs) are promising nanomaterials for realization of strain sensors with high performance. Carbon-nanomaterial-filled composites have been intensively studied as multifunctional material for numerous smart applications. Strain sensors among others have especially attracted a lot of interest [1–11]. Basically, the induced strain alters the electrical resistance of a randomly distributed CNT network. The change of the electrical resistance results from the modification of contact arrangements and the tunneling distance between the CNTs [10, 12–15]. Many scientists reported the use of CNT-filled polymer nanocomposites for strain-sensing [7–9, 11–15]. Among others, polydimethylsiloxane (PDMS) [16–18], polyvinyl alcohol (PVA) [8, 19], and epoxy [8, 20, 21] are considered as promising nanocomposites due to the possibility to modify its strain range by controlling the filler content and filler orientation. However, some difficulties on tailoring the strain sensitivity and mechanical properties of the strain-sensor-based thin films such as the influence of processing parameters and orientation of the CNTs within the polymer matrix were broadly reported [10, 14]. Recently, colloid dispersions and thin-film-based graphene, graphene oxide (GO), and reduced graphene oxide (rGO) films have been widely produced and characterized [22–33]. The investigations pointed out to use them as electrocatalytic materials [34], supercapacitors [35], and transparent electrodes [24, 28, 31]. Nevertheless, their electromechanical properties have not yet been well addressed. GO can be principally used as a dispersant to suspend the agglomerated CNTs in water and to develop a new solution-processing strategy for making GO:CNT hybrid nanocomposites. Compared with pristine multi-walled carbon nanotubes (MWCNTs), such a nanocomposite can be well dispersed in an aqueous solution via the π-π interaction, which stabilizes not only the hydrophobic nanotubes but also provides the MWCNTs with a negative charge [26, 27, 30, 36–38]. Therefore, CNTs dispersed in GO dispersion are expected to have interesting mechanical properties, due to the exceptional mechanical properties of both GO and CNTs.

Properties of the pristine GO and its composites have been widely studied. Wang et al. prepared transparent conductive films based on rGO:SWCNT using a filtration method. The electrical conductivity was improved by a factor of 4–13 after chemical reduction. Besides the electrical properties, X-ray photoelectron spectroscopy (XPS) was used to determine the content of carbon, oxygen, and other elements in the film before and after the chemical reduction [29]. Suk et al. investigated the mechanical properties of the mono-, double, and triple layer of GO using an atomic force microscopy (AFM). The monolayer of the GO had an effective Young’s modulus of 207.6 ± 23.4 GPa [39]. Liu et al. fabricated rGO:CNT using a catalyst-free route method and studied systematically its thermostability, photoluminescence, and electrical properties. The obtained results revealed that introducing CNT to GO improved all the prementioned properties and therefore suggest a high potential application in the field of photonics and electrical devices [40]. Wang et al. reported a synergetic improvement in the electrical and mechanical strength when composite fibers of graphene oxide:single-walled carbon nanotubes (GO:SWCNT) were fabricated by PVA-based coagulation spinning technique [41]. Liu et al. used the first-principle computations to simulate and predict the mechanical properties of the monolayer of GO. He proved that the ordered GO has a Young’s modulus between 380 and 470 GPa, which is higher than the amorphous GO. The change in the Young’s modulus is mainly related to the oxygen coverage, whereas higher coverage leads to lower the Young’s modulus and vice versa [42]. Kotal and Bhowmick made a very detailed investigation of MWCNT chemically bonded to rGO using a wide variety of methods: infrared, Raman, ultraviolet-visible (UV-Vis), X-ray diffraction, XPS, scanning electron microscope (SEM), transmission electron microscopy (TEM), and scanning tunneling microscopy (STM). They found out that the proposed hybrid material exhibits excellent electrical and thermal properties, which made it an excellent candidate for potential energy storage applications such as supercapacitors [43]. Hwang et al. fabricated a transparent thin-film-based rGO with tuned piezoresistivity. The films were reduced using two different methods: (i) temperature and (ii) hydrazine. The manufactured rGO films reduced using hydrazine showed a higher gauge factor (around 8.67) than films reduced using temperature [31]. Trung et al. introduced a new type of flexible strain sensor array based on the rGO field effect transistor (FET), which is able to determine a very small tensile and compression strain of 0.02 %, excellent repeatability up to 10,000 bending cycles, and with fast response and relaxation time [44]. Hwang et al. improved the thermal and mechanical properties of the GO by the addition of the MWCNTs. They stated that amino-functionalized MWCNT bonded to GO exhibits synergetic thermal and mechanical properties compared to the non-functionalized MWCNT [45].

The aim of the present work is to show the feasibility of fabricating the rGO:MWCNT nanocomposite films on a flexible substrate for strain-sensing applications. In this paper, a hybrid nanomaterial GO:MWCNT is synthesized based on the self-assembly of GO and MWCNTs. The nanocomposite films should be highly sensitive to strain, and therefore, its performance should be improved by optimizing the material composition. This can be realized based on the understanding of optical and morphological, as well as the electromechanical, properties of the nanocomposites.

## Methods

**Fig. 1:**
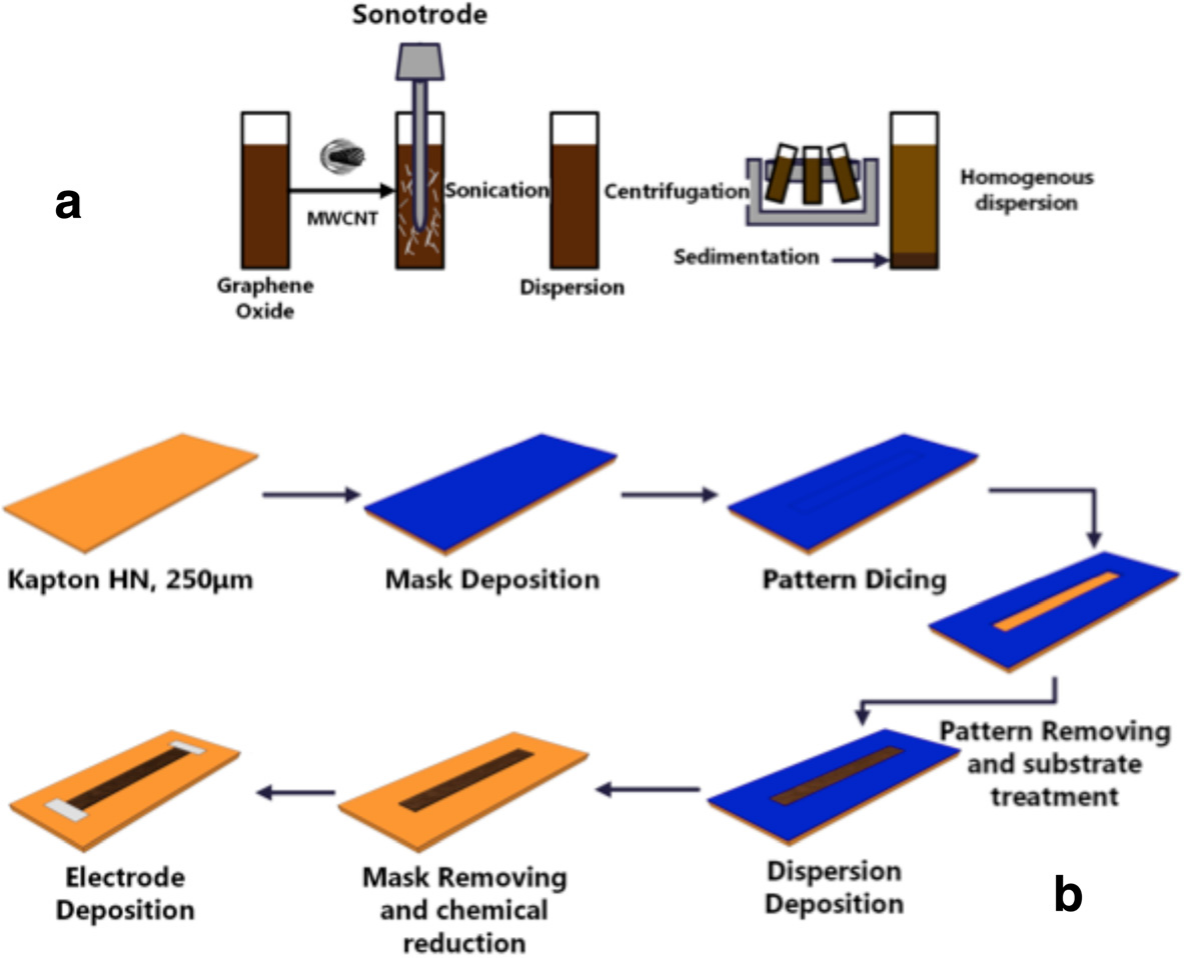
Fabrication process. **a** Aqueous dispersion of GO:MWCNT. **b** Physical liquid deposition of thin film

The GO was purchased from Graphene laboratories Inc., and the MWCNTs from Sigma-Aldrich (Steinheim, Germany), and they were used as received without any further purification, functionalization, or chemical treatment. The GO had a concentration of 0.5 wt.% in water and a composition of 79 % carbon and 20 % oxygen, with a typical flake size of 0.5–5 μm. At least 60 % of the GO flakes had a thickness of one atomic layer. The MWCNTs had a purity of larger than 95 %, outer diameter of 6–9 nm and lengths less than 1 μm. The GO:MWCNT hybrid nanomaterials were prepared by solution mixing of aqueous GO dispersion sheets with different amounts of MWCNTs (0.01, 0.02, and 0.05 wt.%). The mixture was sonicated using a horn sonicator (Bandelin GM 3200) for 30 min at room temperature in an ice bath to avoid overheating. After the sonication process, the dispersion was centrifugated at 5000 RPM for 40 min (Sigma 2-16 PK) to separate bundled CNTs, amorphous carbon, and residual catalytic material. The resulting aqueous solution was deposited using physical solution-casting method on flexible polymer substrates (Kapton HN, 250 μm). Prior deposition, the substrate was cleaned in an isopropanol sonication bath for 15 min, then washed with deionized water (DI-H_2_O) for another 15 min, and finally dried under nitrogen gas. The experimental process used to prepare dispersion and thin film deposition is illustrated in Fig. 1. The final thicknesses of the films were determined by the deposited amount of the dispersion. Using an amount of 62.5 μl, a thickness of 5.8 um ± 16 nm was measured (Veeco Diktak 150).

All the prepared thin films had a footprint area of 2.5 × 0.5 cm^2^. Three films or more from each dispersion were taken into account to ensure good reproducibility and dispersion quality. The fabricated films were reduced using hydro-iodic (HI) acid for 5 min, in order to increase the film conductivity. Longer time leads to reduce the adhesion between the deposited film and the substrate and no big change in the film conductivity were observed. The absorption measurements were carried out with a Lambda 900 UV-Vis/NIR spectrometer from PerkinElmer in the wavelength range of 400–1300 nm. As the dispersion had a very dark color, a dilution process was necessary to ensure good transparency of the dispersion. All the dispersions were diluted in DI-H_2_O with 50:1 (H_2_O:dispersion), and the measurements were performed using a quartz cuvette with 10-mm thickness. The morphological characterization for prepared films was carried out by a scanning electron microscope (SEM) (Nova NanoSEM 230). The ohmic resistance of rGO:MWCNT films was measured with four-point probe method (Keithlay 2602 dual-channel source meter), and then, the DC-electric conductivity was calculated using the following equation:

1 $$ \sigma =\frac{L}{A\times R} $$

where *σ* is the thin film conductivity and *R* is the ohmic resistance; A and L are cross-section area and the thin film length, respectively.

For strain measurements, the samples were stretched using a tensile-compressive cyclic (TCC) machine (Inspekt 10 table from Hegewald & Peschke, Meß- und Prüftechnik GmbH). The setup for the strain measurement is shown elsewhere [9]. The range of the applied force was varied from 0 to 110 N with a step size of 10 N for loading and unloading at a speed of 1 mm/min. At each step force, the I-V characteristics were measured by applying voltage from −0.5 to 0.5 V and the resulting current was recorded by a Keithley 2602 SourceMeter (Keithley Instruments Inc., Cleveland, OH, USA) connected to a host computer through GPIB/USB cable and coupled with the TCC.

## Results and Discussion

### Optical Investigation

**Fig. 2:**
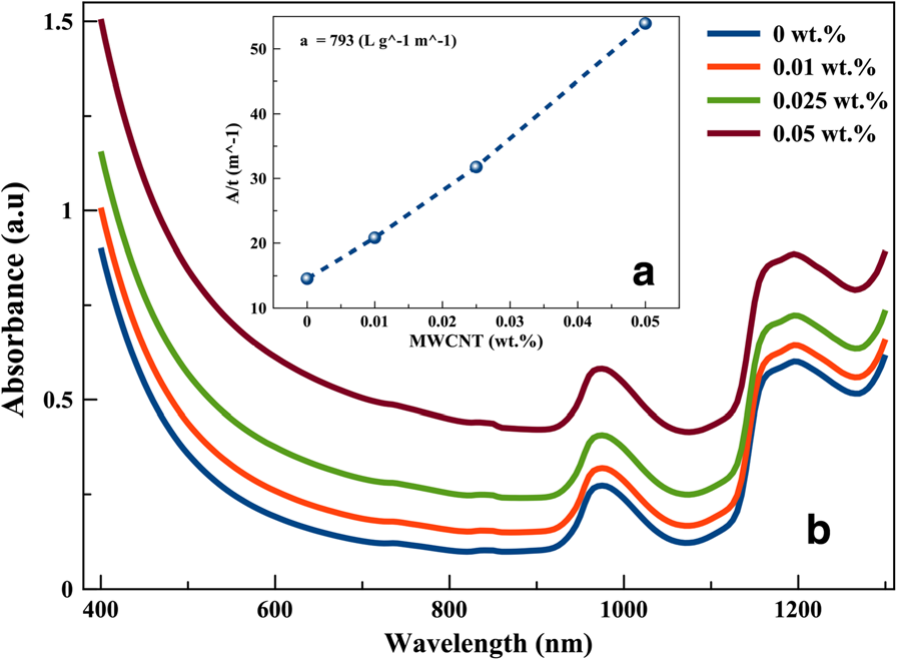
UV-Vis spectra of MWCNT dissolved in GO. (*a*) At different concentrations. (*b*) Absorbance coefficient at 600 nm

The UV-Vis spectra (Fig. 2(a)) show two peaks at 970 and 1200 nm which are attributed to the asymmetric stretch of S(OH)_2_ and its first overtone [46]. From Fig. 2(a), it can be clearly seen that the absorbance (*A*) increases with the amount of MWCNT content dissolved in GO. The Lambert-Beer law (Eq. 2) was applied to determine the absorption coefficient (*α*) at the absorbance of 660 nm (Fig. 2(b)).

2 $$ A=\alpha \times t\times C $$

where *A* is the absorbance, *α* is the absorption coefficient, *t* is the cuvette thickness, and *C* is the concentration.

A linear relationship between the absorbance and the MWCNT concentration (*C*) was extracted, while the absorption coefficient (*α*) was calculated to be approximately 800 L/(g m).

**Fig. 3:**
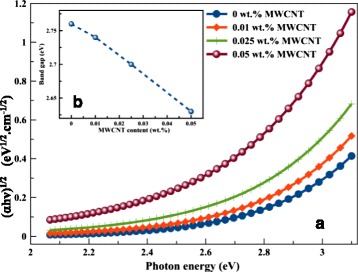
Tauc plot of different MWCNT content dissolved in 0.5 wt.% GO

Considering an indirect band gap of 2.76 eV for graphene oxide, the optical band gap of the hybrid nanocomposite was calculated from a Tauc plot, as depicted in Fig. 3. It can be seen that the increase of the MWCNT content in the GO results in a linear decrease of the band gap due to the partial recovery of the π-conjugation system [36–38]. The band gap decreases from 2.76, 2.74, and 2.7 eV (for 0, 0.01, and 0.02 wt.% MWCNT, respectively) to 2.63 eV (0.05 wt.% MWCNT).

### Morphological Investigation

**Fig. 4:**
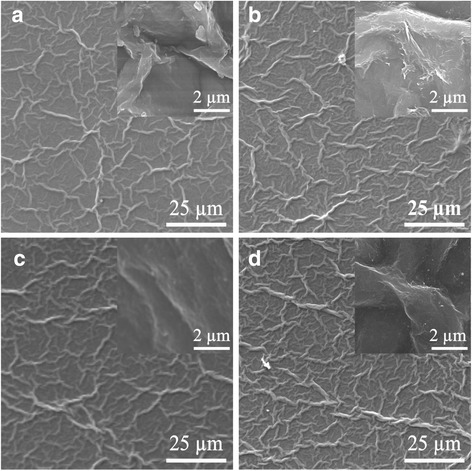
SEM images of **a** 0.5 wt.% rGO, **b** 0.01 wt.% rGO:MWCNT, **c** 0.025 wt.% rGO:MWCNT, and **d** 0.5 wt.% rGO:MWCNT

Morphological study of rGO:MWCNT was conducted using SEM, as it is shown in Fig. 4. The image shows that the graphene layers were coated over the whole area with CNTs and thus confirms the good dispersion quality of MWCNTs in GO aqueous colloids. The wrinkles on the surface of the samples are due to the structural changes with the recovery of the conjugated system, as well as the removal of the hydroxyl and epoxide functionalities from the GO introduced by the chemical reduction [26, 36, 38]. The increase of the MWCNTs will lead to the formation of a dense physical contact network.

### Electrical Investigation

**Fig. 5:**
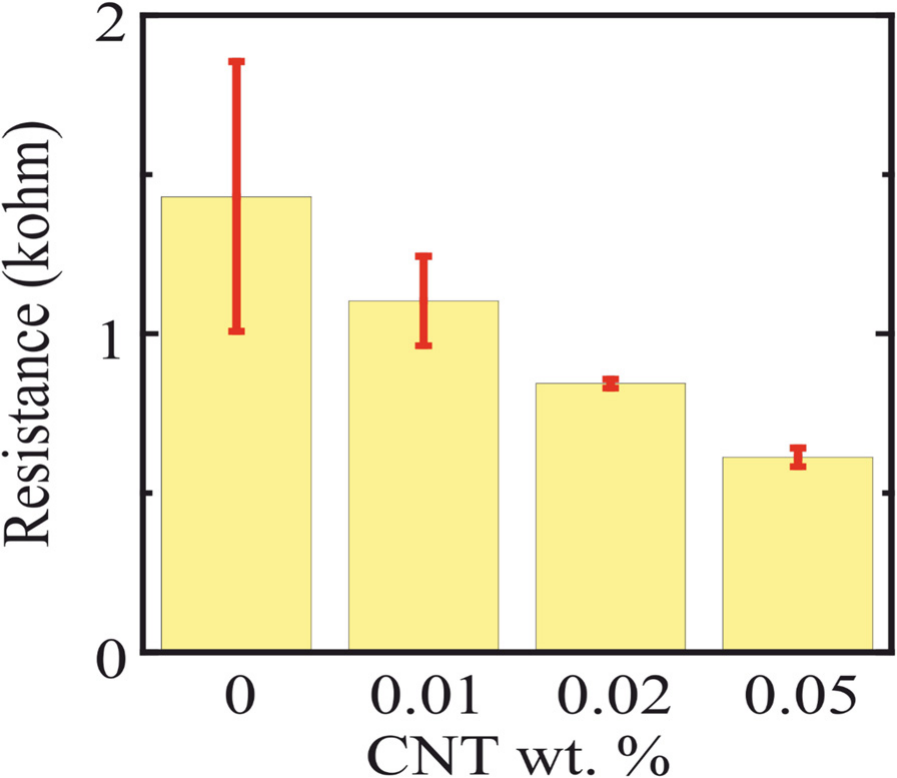
Chemically reduced thin film DC-ohmic resistance as a function of the MWCNT content dispersed in 0.5 wt.% GO

Before reduction, the thin films have an insulating behavior due to the non-stoichiometric chemical composition of the host GO matrix and the presence of the oxidant in the GO that will prevent the formation of percolation paths among the sp^2^ carbon clusters [28]; therefore, the resistances of the films were >50 MΩ. After the chemical reduction, it is well observed that the electrical conductivity of the thin films increased with the amount of MWCNT content (Fig. 5); this is referred to as the high conductivity of MWCNTs and the rGO [47, 48]. The increase in the conductivity of thin films based on rGO:MWCNT hybrid nanomaterial is a result of the strong π-π interfacial coupling between the MWCNT and the rGO that promotes more mobile charge carrier delocalization between the electronic densities of both [28]. However, the thin films were not optically transparent. For the rGO, the thin films had an electrical conductivity of 4.82 × 10^2^ S/m. In the case of rGO:MWCNT, the conductivities were measured to 6.27 × 10^2^, 8.21 × 10^2^, and 1.13 × 10^4^ S/m for 0.01, 0.025, and 0.05 wt.% MWCNTs, respectively (Supplementary data). Besides the improvement in the conductivity by the addition of the MWCNTs (Fig. 5), the thin film reproducibility improved significantly which is well remarked by the decrease in the standard deviation as the MWCNTs content increases.

### Electromechanical Investigation

**Fig. 6:**
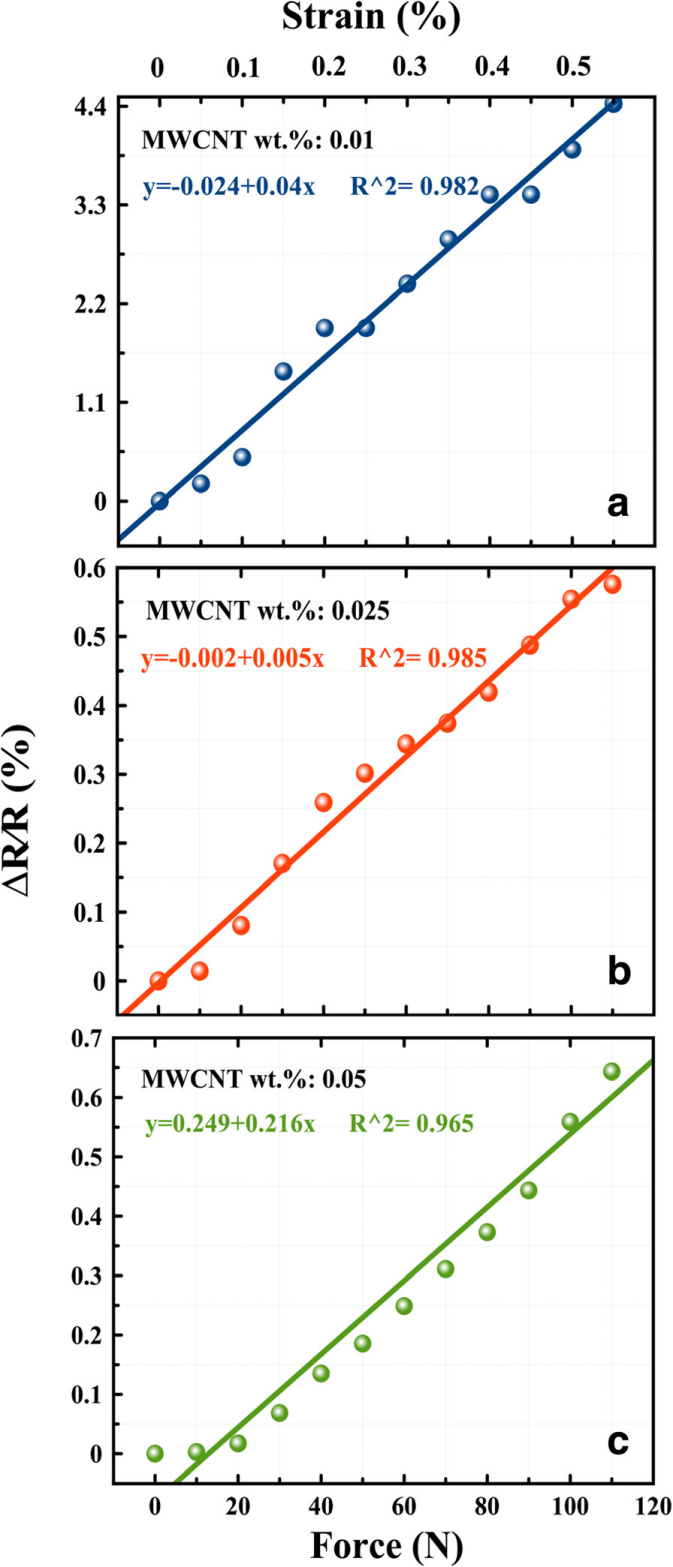
Resistance-strain relationship of rGO:MWCNT under tension cycle. **a** 0.01 wt.% MWCNT, **b** 0.02 MWCNT, and **c** 0.05 wt.% MWCNT

The piezoresistive performances of the rGO:MWCNT films are shown in (Fig. 6) as the normalized change in resistance-strain relationships under an axial tension cycle for samples having 0.01, 0.02, and 0.05 wt.% of MWCNT dissolved in 0.5 wt.% GO. The correlation coefficient (*R*^2^) was ranging between 0.982 and 0.965 for 0.01 and 0.05, respectively, which indicates a high linearity. The strain sensitivity of the hybrid nanocomposites was characterized with the gauge factor *S* using the following equation:

3 $$ S=\frac{\left(\varDelta R/{R}_0\right)}{\varepsilon } $$

where Δ*R* and *R*_0_ are the change of the sensor’s resistance and initial resistance (at *ε* = 0), respectively.

The sensitivity of the thin films decreased with increasing MWCNT content, which is in agreement with the previous works [10, 14]. The average strain sensitivity was 8.5 for 0.01 wt.% and 1.23 for 0.05 wt.% MWCNTs (Supplementary data). The piezoresistivity of the rGO:MWCNT thin films under strain could be explained as a superposition of the following aspects: (i) For rGO, the increase of the resistance is mainly due to the elongation of the bond length between the C atoms in the benzene ring under strain [39, 42]; (ii) For the MWCNTs, the reorientation and the change in the tunneling distance between the adjacent CNTs; and (iii) The change in the number of tunneling contacts formed between the rGO and MWCNT within the conduction network significantly change the total thin film resistance under strain.

## Conclusions

In this paper, we demonstrated the feasibility of realization of rGO:MWCNT nanocomposite films on a flexible substrate for potential use in strain sensor applications. The optical, morphological, and electromechanical properties were investigated for the thin films with different amount of MWCNTs. The UV-Vis spectra show an increase in the absorption as the MWCNT content increases and the tunability of the GO band gap as a function of the MWCNT loading. Electron microscopy results show that the MWCNTs are well dispersed in the GO, forming a uniform dense network. The dependency of the MWCNT amount on both the electrical resistivity and the mechanical properties was investigated. Lower MWCNT content in the GO matrix shows higher sensitivity due to the higher tunneling effect between the neighboring tubes and the shrinkage of the rGO band gap. The reached results are promising for the realization of strain sensors with a high sensitivity reaching a gauge factor of 8.5. We claim that further optimization and improvement of the hybrid composite could be possible to achieve better electrical and mechanical properties in the field of strain-sensing application.

## Additional file

Additional file 1:
**Supplementary data.** The supplementary tables show the DC-electrical resistance of the thin-film-based rGO:MWCNT and the resistance-strain relationship of the thin-film-based rGO:MWCNT.
