# Supplementary material for: Electromechanical Behavior of Chemically Reduced Graphene Oxide and Multi-walled Carbon Nanotube Hybrid Material
Source: Nanoscale Res Lett. 2016 Jan 5;11:4. doi: 10.1186/s11671-015-1216-5 (PMC4701710; doi:10.1186/s11671-015-1216-5)
Supplement: Additional file 1: — Supplementary data. The supplementary tables show the DC-electrical resistance of the thin-film-based rGO:MWCNT and the resistance-strain relationship of the thin-film-based rGO:MWCNT. [file 11671_2015_1216_MOESM1_ESM.docx]

**Supplementary data:**

Title: Electromechanical Behavior of Chemically Reduced Graphene Oxide and Multi Walled Carbon Nanotubes Hybrid Material

Abderrahmane Benchirouf, Christian Müller, Olfa Kanoun

Chair of Measurement and Sensor Technology, Technische Universität Chemnitz, 09126, Chemnitz, Germany

1. **DC-Electrical resistance of the thin films based rGO:MWCNT:**

| **Sample Naming** |  | **Electrical DC-resistance (Kohm)** | **Average value (Kohm)** | **Standard deviation (%)** |
| --- | --- | --- | --- | --- |
| **0.5 wt.% GO** | Sample#1 | 2.01 | 1.43 | 86.39 |
|  | Sample#2 | 1.272 |  |  |
|  | Sample#3 | 1.01 |  |  |
| **0.01 wt.% MWCNT** | Sample#1 | 1.3 | 1.10 | 15.60 |
|  | Sample#2 | 0.984 |  |  |
|  | Sample#3 | 1.024 |  |  |
| **0.025 wt.% MWCNT** | Sample#1 | 0.845 | 0.84 | 2.08 |
|  | Sample#2 | 0.86 |  |  |
|  | Sample#3 | 0.825 |  |  |
| **0.05 wt.% MWCNT** | Sample#1 | 0.576 | 0.61 | 5.96 |
|  | Sample#2 | 0.649 |  |  |
|  | Sample#3 | 0.612 |  |  |

| **Force (N)** | **DC-Resistance (Kohm)** | | | |
| --- | --- | --- | --- | --- |
|  | **0.0 wt.% MWCNT** | **0.01wt.% MWCNT** | **0.025 wt.% MWCNT** | **0.05 wt.% MWCNT** |
| **0** | 1423.300018 | 1101.853394 | 844.4807283 | 612.7826506 |
| **10** | 1377.52669 | 1103.974461 | 844.6013201 | 612.7998698 |
| **20** | 1318.677504 | 1107.224929 | 845.161726 | 612.8885149 |
| **30** | 1165.27575 | 1123.218793 | 846.6015435 | 613.3091709 |
| **40** | 1010.352339 | 1144.895793 | 848.7944955 | 614.1383036 |
| **50** | 857.9305851 | 1166.991136 | 851.3547992 | 615.2784514 |
| **60** | 718.9973062 | 1195.232322 | 854.2854178 | 616.8096333 |
| **70** | 608.8397289 | 1230.069758 | 857.4841187 | 618.7283431 |
| **80** | 511.6567314 | 1272.06803 | 861.0812646 | 621.0362616 |
| **90** | 425.0281302 | 1315.500249 | 865.2781747 | 623.7901227 |
| **100** | 354.0314313 | 1367.052073 | 870.0728541 | 627.2779205 |
| **110** | 295.400286 | 1427.587873 | 875.0828206 | 631.3142657 |

1. **Resistance-Strain relationship of the thin films based rGO:MWCNT:**
